# Differences in students’ scholastic well‐being induced by familial and scholastic context

**DOI:** 10.1111/bjep.12484

**Published:** 2021-12-26

**Authors:** Ramona Obermeier, Juliane Schlesier, Michaela Gläser‐Zikuda

**Affiliations:** ^1^ Johannes Kepler University Linz Austria; ^2^ Department of Educational Sciences Primary Education (Chair) Faculty of Arts and Humanity University of Greifswald Germany; ^3^ Friedrich‐Alexander‐University Erlangen‐Nuremberg Germany

**Keywords:** individual differences, instructional quality, parent–child interaction, scholastic well‐being

## Abstract

**Background:**

Since scholastic well‐being is connected with intrinsic motivation, positive emotions and effective learning, it is highly relevant for educational research. It is influenced by a variety of individual and contextual determinants and differs for several groups of students with respect to their environmental conditions.

**Aims:**

Up to now, there has been neither approach in answering questions about group‐differences between students with high or low levels of scholastic well‐being nor in defining variables that are most different for these groups. The current study addresses this research gap by investigating differences in familial and scholastic aspects in two distinct groups of students (extreme high or low level of scholastic well‐being).

**Sample and Method:**

Self‐report questionnaires from *N* = 852 fifth graders were evaluated using the multivariate analysis of variance (MANOVA) and a discriminant analysis.

**Results:**

Results of the discriminant analysis show that attainment of academic educational track, good classroom‐management, positive social climate in class and high clarity of instruction, as well as low parental pressure on performance are characteristics that classify students with an extreme high level of scholastic well‐being. Therefore, those variables can be used to divide students into disjoint groups without having any information about their actual scholastic well‐being.

**Conclusion:**

Firstly, it can be deduced from the findings that measures within the schools to promote scholastic well‐being should start with the improvement of instructional quality and social climate. Second, reduction of parental pressure on performance as well as the implementation of successful cooperation between families and schools is vital.

## Background

Scholastic well‐being, as a domain‐specific facet of subjective well‐being, is an important foundation for successful learning (Borgonovi & Pál, 2016; OECD, 2015, 2018; Putwain, Loderer, Gallard, & Beaumont, 2020). Thus, it is considered to be of high importance in educational research. The development of scholastic well‐being is influenced by several individual and contextual aspects within the school and family (Kutsyuruba, Klinger, & Hussain, 2015; Putwain et al., 2020; Suldo & Fefer, 2013). Variables regarding students’ social backgrounds and environmental conditions in schools lead to differences in cognitive and affective development (Baumert, 2006; van Ophuysen, 2009). The impact of social origin on interests, preferences and desires is a well‐known phenomenon. Several studies claim that inequalities in socio‐emotional development and health are linked to familial background (Börsch‐Supan et al., 2019). There is also some evidence that scholastic well‐being is induced by different environmental conditions in schools, as well as in families.

Many studies (Choi, 2018; Morinaj & Hascher, 2019) underpin the effect of the scholastic environment on students’ scholastic well‐being, especially the quality of instruction and the social climate in class. In contrast, less is known about the impact of familial conditions on scholastic well‐being. A small number of studies addressing predictors of scholastic well‐being in extracurricular contexts found some familial conditions to be influencing factors (Hascher & Hagenauer, 2020). However, those studies focus mainly on the impact of socio‐economic status, while neglecting aspects of social interaction in families. Thus, previous studies have not aimed to explain contextual differences (in both scholastic and familial environments) between students who exhibit extremely high or low levels of scholastic well‐being.

### Scholastic well‐being

Scholastic well‐being is a multidimensional construct reflecting both cognitive and affective facets of scholastic experiences (Hascher, 2007; Putwain et al., 2020). Cognitive aspects include thoughts about a student’s own abilities, classmates, teachers and school as an institution. The affective dimension comprises feelings about teachers and peers, or scholastic requirements (Putwain et al., 2020).

There is consensus about the highly subjective nature, perception and determination of well‐being in general (Diener, 1984) – in which contextual factors play an important role – and scholastic well‐being is no exception (Hascher, 2007). Scholastic well‐being includes positive and negative components and is strongly related to personal growth, intrinsic motivation and basic needs (Deci & Ryan, 1996; Hascher, 2003, 2007; Su, Tay, & Diener, 2014). When self‐evaluations of positive affect outweigh the extent of negative affect, scholastic well‐being is generally rated highly (Diener, Oishi, & Tay, 2018; Hascher, 2003, 2007). A low level of scholastic well‐being impairs the learning process, social interactions and health, and is associated with higher levels of absence and dropout (Morinaj & Hascher, 2019; Putwain et al., 2020). Thus, scholastic well‐being is an aspect of growing interest for educational science, school administration and policymakers (Bonell et al., 2014; OECD, 2018; Putwain et al., 2020).

Due to the fact that well‐being is considered to be domain‐specific, previous research on scholastic well‐being has focused mainly on the effect of the scholastic environment (Grigoryeva & Shamionov, 2014; Hascher, 2003; Kutsyuruba et al., 2015). Referring to self‐determination theory (Deci & Ryan, 2002), (scholastic) environments can be classified by their potential to fulfil a student’s basic needs for autonomy, competence and social relatedness, which in turn affect the development of intrinsic motivation, positive emotions and well‐being. Self‐determination theory (Deci & Ryan, 2002) provides a basis for the classification of both scholastic and familial contexts. Based on the assumption that positive social relationships – in and out of school – are vital for the development and maintenance of scholastic well‐being (Hascher, 2003), parents as well as peers and teachers are an important source of social support for students (Suldo & Fefer, 2013). With respect to ecological theories (Bronfenbrenner, 1979), human development is processed simultaneously in different social systems (i.e., family, school or neigbourhood – Ditton, 2006). In particular, there are various theoretical and empirical models that pay attention to extracurricular environments (Hascher, Morinaj‐Turkina, & Waber, 2018). Although we might presume that the family can buffer any negative effects of schooling, and vice versa, little is known about these compensatory effects, particularly with respect to the fulfilment of students’ basic needs in terms of autonomy and competencies. For example, conducive scholastic or familial environments facilitate development of positive self‐concept and adaptive attribution styles. Students with a more positive self‐concept, who tend to attribute success to internal aspects (e.g., high competence), are less vulnerable to negative experiences in another context (Gizir & Aydin, 2009; Masten, Herbers, Cutuli, & Reed, 2009). Those students are often classified as resilient, and thus more resistant to adverse conditions.

Some studies have investigated the mitigating effect of school with respect to discrepancies in the health of children and adolescents from different social milieus (Hascher & Winkler‐Ebner, 2010; Obradović & Armstrong‐Carter, 2020), confirming that the health status of students converges during their school career. Despite findings with respect to the huge impact of familial socialization on personality development (Bronfenbrenner, 1979; Ditton, 2006), the studies mentioned tend to ignore the potential compensatory effect of good familial conditions on scholastic well‐being.

### Scholastic influences on scholastic well‐being: current research findings

One important aspect that should be mentioned when discussing scholastic influences on scholastic well‐being is the educational track. The German educational system is highly selective: Students have to choose their future educational track at an early stage (mostly after fourth grade). The choices are between the lower educational track (German Haupt‐ or Mittelschule); the middle educational track (German Realschule) which is orientated towards vocational preparation; or the higher educational track (German Gymnasium) that prepares them for an academic career. Research in German‐speaking countries shows that the cognitive and affective development of children depends on the chosen educational track. Lower educational tracks exhibit lower initial levels and a decrease in scholastic well‐being (van Ophuysen, 2009), whereas higher educational tracks tend to exhibit higher levels of scholastic well‐being (Herke, Rathmann, & Richter, 2019).

A second aspect of structural conditions of schooling is single‐sex education (SSE). Research shows contrary results, but indicates higher scholastic self‐concept and scholastic well‐being of girls in homogenous groups (Crawford‐Ferre & Wiest, 2013; Else‐Quest & Peterca, 2015; Herwartz‐Emden, Schurt, & Waburg, 2012).

Further studies that address the school environment imply that students who perceive school as being untidy and unstructured are lacking in positive emotions and positive cognitions, and thus in scholastic well‐being (Kutsyuruba et al., 2015). Since the effects of spatial‐structural conditions of schools are rather low, it is presumed that processual aspects of school life (e.g., the climate in class) more strongly affect the scholastic well‐being of students (Hascher, 2003, 2007). Consequently, class climate is a topic of high educational interest and an outstanding predictor of affective outcomes in education (Choi, 2018; Gase et al., 2017). It is a multidimensional aspect that includes feelings and attitudes towards school evoked by experiences during a student’s school life (e.g., social relationships, and methods of teaching and learning – Gase et al., 2017; Kutsyuruba et al., 2015; Zullig, Koopman, Patton, & Ubbes, 2010). Social relatedness is considered a psychological need and positive social relations are a necessary precursor to children’s willingness to explore, and therefore the basis of other psychological needs, such as feelings of competence and autonomy (Ryan & Deci, 2002). Accordingly, a social climate that provides safety, responsivity and emotional warmth is essential for effective learning and scholastic well‐being (Lee & Yoo, 2015; Morinaj & Hascher, 2019).

Numerous studies which address student–teacher interaction (apart from other aspects of classroom climate) point out that teachers’ solicitousness is a predictor of a positive attitude towards school (OECD, 2015, 2018) and scholastic well‐being (Grigoryeva & Shamionov, 2014; van Petegem, Aelterman, Rosseel, & Creemers, 2007). Solicitousness is defined as the combination of social competencies and teaching ethos to offer emotional responsivity, safety, justice and support in addressing both scholastic and non‐scholastic problems (Gläser‐Zikuda & Fuß, 2008).

Other characteristics of teaching (e.g., classroom management and clarity of instruction) are also associated with positive emotional experiences and scholastic well‐being (Hagenauer & Hascher, 2011). The second aspect addressed by the current study is the impact of familial background on scholastic well‐being.

### Familial influences on scholastic well‐being: current research findings

Socio‐economic status and cognitive facilitation in children’s homes are associated with health, well‐being and educational outcomes (Hattie, 2010; OECD, 2015, 2018; Rimkute, Hirvonen, Tolvanen, Aunola, & Nurmi, 2012). Lee and Yoo (2015) found that material resources affect the level of a child’s subjective well‐being, even if the statistical power of such effects found in the literature is relatively low (Lee & Yoo, 2015). However, as soon as a child’s basic needs are met, the effects of income, socio‐economic and marital status of parents mostly disappear. Instead, processual aspects of family life come to the fore, which include parenting style, communication structures, collective activities, and parental involvement in school and homework (Epstein, 2011; Suárez et al., 2016). Those aspects derive from structural conditions. Familial socialization – that includes unintentional approaches of parents to modifying children’s behaviour – is considered to be the main contributor to the development of specific beliefs, values and attitudes towards formal education and learning in school (Lazarides & Watt, 2017; Rimkute et al., 2012; Simpkins, Fredricks, & Eccles, 2012). Furthermore, secure attachment between children and parents and a positive parent–child relationship are fundamental premises for further relationships, successful personality growth, and well‐being in general (Goswami, 2012; Suldo & Fefer, 2013). A safe, inviting and comfortable home environment, as well as harmonious interactions and joint activities, are key factors of well‐being during adolescence (Joronen & Astedt‐Kurki, 2005; Suldo & Fefer, 2013). Regarding students’ learning, high parental involvement in school is associated with positive affective outcomes (Suldo & Fefer, 2013). Students who report higher parental interest in their scholastic activities score higher on items regarding life satisfaction and academic motivation (OECD, 2018). More frequent parent–child activities are associated with higher levels of well‐being (Lee & Yoo, 2015). On the contrary, high pressure on performance, little interest in scholastic aspects and intrusive support are associated with lower scholastic well‐being (Suldo & Fefer, 2013).

Based on these findings, it is clearly necessary to take familial background and familial support into account when investigating the affective development of children in the school environment. Some of the studies discussed recommend approaches that contribute to understanding the mutual impact of family and school on the affective development of children. Nevertheless, there have been no endeavours that seek variables on the contextual side which differ significantly between students with high or low scholastic well‐being. A targeted focus on these extreme groups is particularly interesting as it provides a direct comparison, as well as insights into the perception of contextual aspects for both groups.

### Aims and hypotheses

Students who have few opportunities to meet their psychological needs in school (and in their family) are expected to report lower levels of scholastic well‐being. Yet it could also be possible that students with high or low levels of scholastic well‐being differ in their perceptions of environmental conditions. The current study aims to explore differences in contextual conditions for two groups of students characterized by extreme high or low levels of scholastic well‐being. The two groups are compared with respect to their scholastic and familial environments, in order to (1) accurately predict group membership by the chosen contextual variables; and (2) find the linear combination that most reliably classifies students in one of the two groups.

Based on our knowledge about the mutual impact of familial and scholastic aspects on scholastic well‐being, we included both environments in the analyses equivalently. We hypothesized that students with extreme high or low levels of scholastic well‐being differ significantly in their perceptions of structural (educational track, single‐sex education, school equipment; socio‐economic, migration and educational background of their parents) and processual (quality of instruction, teachers’ solicitousness; parental involvement) contexts. To reveal those differences in both environments, we performed a MANOVA and an affiliated discriminant analysis. Discriminant analysis enables determination of the probability of correctly assigning students to a group (extreme high or low scholastic well‐being) via contextual variables; this has *particular relevance for the practical implication of the results in schools* in order to positively influence scholastic well‐being in the long term through other variables that are easier to influence via teacher training courses (e.g., quality of instruction).

Prior research has shown that students who follow the academic (higher) educational track are associated with higher scholastic well‐being than students in the medium educational track. Furthermore, we anticipated that students who report a lower level of scholastic well‐being are taught in schools with lower quality of instruction and more impersonal teacher–student relationships.

Concerning familial aspects, we postulated that structural aspects of families (e.g., low access to education‐related resources) would be more prominent in the group of students with lower levels of scholastic well‐being. In addition, we expected that a negative family climate (operationalized by high pressure on performance and low levels of adaptive support regarding homework) would contribute to a low level of scholastic well‐being.

## Method

### Study design

This study was part of a cross‐sectional study in schools in Southern Germany, supported by the Catholic Foundation. The sample included schools on the medium (German Realschule) and the academic educational track (German Gymnasium). In fall 2017, we surveyed *N* = 852 fifth graders using the online tool LimeSurvey, and paper‐and‐pencil questionnaires to collect data from their parents. Information about migration background, educational attainment and marital status of the parents was retrieved from the parent questionnaire, while all other aspects were taken from the students’ data. The student and parental data were matched using an individual family code.

### Measures

Data on structural aspects of the school were collected from the student questionnaire and coded as follows: educational track (1 = Gymnasium, 0 = Realschule); single‐sex school (1 = co‐educational school, 0 = mono‐educational school). The variables taken from the parents’ questionnaire were also dichotomous and were coded as follows: educational background (1 = low; 0 = medium or high); migration status (1 = migration, 0 = no migration); and marital status (1 = marriage/long‐lasting partnership, 0 = single parent). Educational background was measured by the ISCED‐97 level.^1^ Based on that classification, medium educational background was assumed if vocational training had been completed, while high educational background implies holding an academic degree. Further insights into education‐related resources at home were operationalized using a 4‐point Likert scale which classified the number of books in the home (1 = <50, 2 = 50–100, 3 = 100– 150, 4 = more than 150).

All categorical variables (scholastic well‐being, quality of instruction, caring of the teacher, parent–child interaction) were assessed via student ratings on a 5‐point Likert scale (1 = not agree at all; 5 = absolutely agree). Scholastic well‐being was captured using a global scale based on Hascher’s (2007) instrument. School‐related aspects concerning the quality of instruction (e.g., classroom management, climate in class and clarity of instruction) were measured using an instrument taken from Lenske (2013), while teachers’ solicitousness was assessed using the LASSO scales (Saldern & Littig, 1987).

In order to gather information about their familial background from the students’ perspective – especially parent–child interactions related to school – we used the scales of Wild et al. (2001). All scales within the students’ questionnaire revealed sufficient to very good internal consistencies according to Cronbach`s alpha based on these data (see Table 1). As information retrieved from the parents’ questionnaire included only single items with respect to socio‐demographic aspects, it is not displayed in the table.

**Table 1:** Scales in the students’ questionnaire showing examples, number of items and internal consistencies

Author	Scale	Item	Number of items	Cronbach’s α
Hascher (2003)	Scholastic well‐being	“I like to go to school.” “School makes sense to me.” “During the last few weeks I was happy because my classmates accepted me.” “During the last few weeks I worried about handling the school reality”	28	.89
Wild et al. (2001)	Pressure on performance	“In case of a bad grade my parents give me a hard time”	6	.80
	Homework support	“My parents are happy for me if I succeed in school”	6	.86
Lenske (2013)	Classroom management	“In school I can learn without being disturbed”	5	.72
	Social climate	“Students are friendly to each other”	6	.86
	Clarity of Instruction	“Mostly I understand the topics”	5	.83
Saldern and Littig (1987)	Teachers’ solicitousness	“The teacher cares about our problems”	7	.82

### Sample

The data of *N* = 852 fifth graders (age *M* = 10.19, *SD* = 0.44) with attending German secondary schools on the medium track (50.9%) or academic track (49.1%) were analysed. The sample includes a higher percentage of female students (78.7%) because nearly a third of the schools in the sample were all girls’ schools. Thus, 51.7% of the students attend mono‐educational classes. Additionally, 26.2% of the students have a migration background and the majority (97.2%) are from families with medium to high educational backgrounds. Slightly more than half (54.9%) of the students reported that they have at least 100 to more than 150 books at home. Table 2 shows the composition between the two groups.

**Table 2:** Students’ characteristics in both groups differentiated by the mean of scholastic well‐being

	Low well‐being	High well‐being
	*N* = 426	*N* = 426
	Percentage	
Educational track	60.8% medium track	41.1% medium track
Sex	76.6% girls	80.8% girls
Single‐sex education	49.3% SSE	54% SSE
Migration	30% migration	22.3% migration
Single parent	8.7%	7.3%
Low educational background	2%	3.4%
High educational background	46.2%	56.1%
Number of books	21.6% more than 150	31.2% more than 150

### Data analysis procedures

All calculations were carried out using SPSS 26 (2020). Missing values ranged from 13.3% to 27.8%, and Expectation‐Maximation (EM) estimation was conducted to complete the dataset. With respect to the intended analysis, this approach has proven to be adequate because of its reliable estimation of variances and covariances when imputing data for large sample sizes under missing at random (MAR) conditions (Baltes‐Götz, 2013).

In order to compare extreme groups which are more prone to producing distinct results, the sample was divided by doing a tertile split. This approach seemed to be the most promising, since we aimed to explore the extent to which students with extreme high or low scholastic well‐being differ in terms of contextual circumstances. Therefore, the middle third (*N* = 419), which contained students who reported moderate scholastic well‐being, was excluded from the analyses. This approach was taken for two reasons: Firstly, since individual aspects (e.g., self‐efficacy, achievement emotions, personality traits) also influence scholastic well‐being – which might mitigate the effects of context – the comparison of two extreme groups is purposeful in estimating group differences. Secondly, the group of students with very low scholastic well‐being is of particular interest, as they are at higher risk of dropping out of school (Morinaj & Hascher, 2019).

Correlations between dichotomous and metric variables were calculated using the point‐biserial Pearson‐*r* correlation coefficient, which is equivalent to the biserial Eta coefficient in cases with 0/1 dummy coding (Bortz & Weber, 2005). Coefficients with values in the range of .31 < *r* < .50 were considered to be of medium strength.

Since the structure of the data is nested (students in classes), the intraclass correlation was calculated in advance in order to decide if multilevel analysis was necessary. The relatively low *ICC* (*ICC* =.04), and the individual centred approach that aimed at classifying each student based on their subjective perception of contextual variables, clinched the decision against a multilevel approach (discussed later). Prior to the analysis, the distribution attributes of the variables were checked. To ensure that the intended calculations complied with the assumptions, z‐standardized variables were used for further analyses. Variables which did not follow the required statistical normal distribution were transformed using an inverse transformation. Even though the *Levene Tests* and *Box Test* – which are known to be susceptible (Field, 2018) – showed significant inequality of error variance of the dependent variables, a visual check of Q–Q‐plots implied a statistically normal distribution.

In order to confirm differences between the two groups (with low or high levels of scholastic well‐being) in terms of contextual conditions, an analysis of covariance was computed using the multivariate analysis of variance (MANOVA). MANOVA was applied to test group differences in students’ perceptions of school and teachers’ behaviour (classroom management, clarity of instruction, climate in class, and teachers’ solicitousness), as well as in their conditions at home (pressure on performance, autonomy support, interest in school, books at home). This calculation was controlled for educational track, migration and educational background. Because the group sizes were equal, Pillai’s trace was used and interpreted (Field, 2018).

Discriminant analysis was conducted to estimate exactly how the groups are differentiated, and thus to expand the findings from the MANOVA that reveal significant group differences. MANOVA and discriminant analysis follow different logics as MANOVA uses group membership as the independent variable to reveal differences in several dependent variables, whereas discriminant analysis uses those prior dependent variables to classify subjects into the former independent group. Hence the two approaches switch the variables from dependent to independent, and vice versa, so they complement each other and are often used consecutively (Field, 2018). Furthermore, discriminant analysis enables determination of the probability of correctly assigning students to a group. A stepwise procedure of entering discriminant variables and creating a discriminant function – which maximizes the quotient of variance within and between the groups – was applied. High eigenvalues and canonical correlation coefficient, combined with a low Wilks’ ***λ***, suggested a good fit (Field, 2018).

## Results

### Descriptive statistics

All metric variables were calculated using a 5‐point Likert scale (1 = not agree at all to 5 = absolutely agree), with high values indicating positive perceptions of the mentioned aspect. The students rated the teachers’ solicitousness (*M* = 3.65; *SD* = 0.71) and the quality of instruction from medium to high (classroom management: *M* = 3.60; *SD* = 0.75; social climate: *M* = 4.00; *SD* = 0.76; clarity of instruction: *M* = 3.87; *SD* = 0.74). Perceived parental support was rated in the upper third of the scale (e.g., homework support: *M* = 4.19; *SD* = 0.74), and parents’ interest in scholastic activities was also rated relatively highly (*M* = 3.93, *SD* = 0.71). Perceptions of parental pressure on performance were lower (*M* = 1.90; *SD* = 0.73).

**Figure 1:**
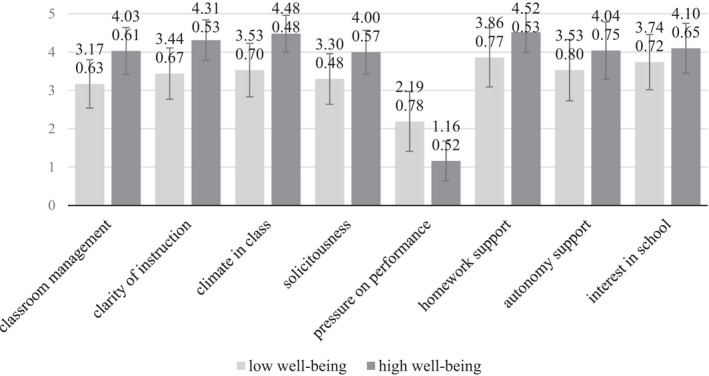
Group means and standard deviations of contextual aspects for students with high or low scholastic well‐being.

Regarding the two subgroups of students with a very low (*M* = 3.28, *SD* = 0.34) or very high (*M* = 4.39, *SD* = 0.19) level of scholastic well‐being, some differences are evident with respect to perceptions of their scholastic and familial environments. On scholastic level there is a more negative perception of several aspects (i.e., clarity of instruction, climate in class) and a more negative assessment of parental behaviour (i.e., pressure on performance) in the group of students with lower scholastic well‐being (see Figure 1).

Correlation analyses reveal positive associations between scholastic well‐being and educational track, migration background and a two‐parent family. However, those correlations are rather low/medium. There are moderate correlations between scholastic well‐being and perceived parental support (e.g., homework support: *r* = .48, *p* < .01), as well as high correlations with the perceived instructional characteristics (e.g., climate in class: *r* = .69, *p* < .01; see Table 3).

**Table 3:** Point‐biserial correlations between the categorical and dummy variables

Scholastic well‐being	
	*r*
1. Scholastic well‐being	
2. School	.18**
3. Single‐sex education	−.08**
4. Gender	−.08**
5. Migration	.10**
6. Number of books^a^	.10**
7. Classroom management	.64**
8. Social climate in class	.69**
9. Clarity of instruction	.65**
10. Teachers’ solicitousness	.56**
11. Parental autonomy support	.34**
12. Parental performance pressure	−.46**
13. Parental homework support	.48**
14. Parental interest in school	.27**
15. Family with two parents	.11**
16. Low educational background	.03**

^a^Because the number of books is ordinal data, we used and report the non‐parametric Spearman’s coefficient.

**p < .01; *p < .05.

### MANOVA

Multivariate analysis of variance was used with group membership as the independent variable, to estimate differences in several dependent variables concerning contextual variables. Pillai’s trace implies a significant difference between the two extreme groups of students, *V* = 0.43, *F*(12, 827) = 53.84, *p* < .01. The MANOVA implies that the students with extremely high or low levels of scholastic well‐being differ in terms of enrolment in educational tracks, *F*(1, 838) = 32.37, *p *< .01, η^2^ = .04 and migration background, *F*(1, 838) = 6.90, *p* < .01, η^2^ = .01. Furthermore, students also differ in their perceptions of classroom management, *F*(1, 838) = 395.75, *p* < .01, η^2^ = .32, social climate in class, *F*(1, 838) = 42.10 *p* < .01, η^2^ = .29, clarity of instruction, *F*(1, 838) = 287.50, *p* < .01, η^2^ = .26 and teachers’ solicitousness, *F*(1, 838) = 268.37, *p* < .01, η^2^ = .24.

Familial aspects are also perceived differently, albeit with a lower effect size. The most powerful differences are found with regard to parental pressure on performance, *F*(1, 838) = 170.61, *p* < .01, η^2^ = .17 and homework support, *F*(1, 838) = 144.35, *p* < .01, η^2^ = .15. Parental interest in school activities, *F*(1, 838) = 54.83, *p* < .01, η^2^ = .06 and autonomy support, *F*(1, 838) = 88.60, *p* < .01, η^2^ = .10 also vary between the groups.

### Discriminant analysis

Finally, discriminant analysis was conducted to investigate the linear combination revealed by MANOVA in more detail. Only the significant variables were used as independent variables to assign the students into the two a‐priori defined groups, which were treated as the dependent variables. The discriminant function has an eigenvalue of .76 (canonical correlation: .66).

Model 6, which demonstrates the best fit (see Table 4), contains the variables *social climate in class, clarity of instruction, classroom management, teachers’ solicitousness, educational track,* and *parental pressure on performance*, all of which were proven to differ significantly between the two subgroups in the MANOVA. The six named variables distinguish the two groups in a statistically significant way, λ = 0.57; χ^2^(6) = 479.34, *p* < .01.

**Table 4:** Number of variables entered into the discriminant analysis and fit of the function

Number of variables/model	λ	Exact *F*			
		*df*1	*df*2	*F* statistic	Sig.
1	.68	1	850	406.97	.000
2	.63	2	849	251.33	.000
3	.60	3	848	186.33	.000
4	.59	4	847	147.08	.000
5	.58	5	846	124.94	.000
6	.57	6	845	107.19	.000

Note

λ = Wilks Lambda, *df*1 = degrees of freedom for parameters, *df*2 = degrees of freedom for participants.

The standardized canonical correlation coefficients indicate that classroom management provides the strongest correlation with the discriminant function (*r* = .41), whereas the other variables correlate rather low with the discriminant function (see Table 5).

**Table 5:** Canonical discriminant function coefficients and structure matrix

	Canonical discriminant function coefficients	Structure matrix
Educational track	.25	.23
Classroom management	.41	.79
Social climate in class	.23	.73
Clarity of instruction	.20	.67
Parental performance pressure	−.30	−.52
Teachers’ solicitousness	.25	.65

These results show that variables which measure several dimensions of perceived instructional quality (e.g., positive climate in class, teachers’ solicitousness, clarity of instruction and classroom management) differ the most between the two extreme groups of students. High evaluation of teachers’ quality of instruction is significantly more common in the group of students with extremely high levels of scholastic well‐being. A higher educational track also appears to be more prominent in the same group of students, while high pressure on performance in parent–child interaction is more common in the group with low levels of scholastic well‐being.

Taken together, 82.5% of the 852 original grouped cases and 81.7% of the cross‐validated grouped cases were correctly classified. Thus, the contextual aspects mentioned are useful for distinct classification of students into two groups with high or low levels of scholastic well‐being.

## Discussion

### Theoretical significance

The findings of the current study support the assumption that students with extreme high or extreme low levels of scholastic well‐being differ significantly in structural characteristics and their perception of processual aspects of scholastic and familial environments. Our MANOVA and discriminant analysis results show that intra‐scholastic aspects (e.g., classroom management, social climate in class, and clarity of instruction) – as well as familial pressure on performance – enable us to differentiate students into two distinct groups with different levels of scholastic well‐being.

Students with more favourable scholastic and familial conditions – meaning higher perceptions of a positive social climate in class and quality of instruction; and lower perceptions of performance pressure from family – form the group of students with a high level of scholastic well‐being that is statistically significantly diverse from the group of students with low scholastic well‐being. Accordingly, students in the group with a higher level of well‐being tend to rate the perceived scholastic conditions more positively, or are in fact located in classes with better conditions (e.g., more positive social climate and higher clarity of instruction). In contrast, students who report a lower level of scholastic well‐being also report lower perceptions in relation to those scholastic aspects.

The MANOVA reveals a significant difference in educational track attained between students with high or low levels of scholastic well‐being. Even the students in the sample who recently achieved the transition to the academic track are more likely to have a higher level of scholastic well‐being, which is in line with prior findings (Herke et al., 2019). The highest effects are caused by clarity of instruction, classroom management, teachers’ solicitousness and social climate in class. In accordance with recent studies (Morinaj & Hascher, 2019), these findings emphasize the major impact of school processual aspects.

Furthermore, the analyses in the present study show that familial pressure on performance is also significant in classifying students. Students with extreme high or low scholastic well‐being differ significantly in their reports of pressure on performance experienced at home: students with high perception of pressure are more likely to belong to the group with a low level of scholastic well‐being (Suldo & Fefer, 2013). Structural aspects of family background (e.g., migration or educational background) are not significant and therefore not appropriate in differentiating groups of students by their level of scholastic well‐being. This supports the assumption that structural aspects have only a slight impact on scholastic well‐being (Lee & Yoo, 2015).

### Practical implications

Students with high or low levels of scholastic well‐being can be classified by investigating their perceptions of their scholastic and familial environments, thus making it possible to divide students into two distinct groups based solely on those characteristics. We posit that ratings of instructional quality, familial conditions and school structural aspects are important factors in estimating the cognitive and affective dimensions of students’ scholastic well‐being.

The benefits of this study’s findings can be found at two levels: Firstly, the findings provide additional information on group differences of students with high or low levels of scholastic well‐being, thus expanding prior studies on predictors of scholastic well‐being. Taking this perspective, it is feasible to estimate the actual state of scholastic well‐being of students, based purely on information provided by contextual variables. Secondly, the findings underpin the notion that data collected in the interests of school improvement may also be used to classify students into groups with high or low levels of scholastic well‐being. Thus, interventions can be carried out at an early stage and without further investment in collecting additional data.

Finally, the results of the study also strengthen the importance of parent–child interaction for students’ scholastic well‐being. Families and schools both contribute to students’ scholastic well‐being and each context is able to buffer negative effects from the other (Kirk, Lewis‐Moss, Nilsen, & Colvin, 2011; Wong, Chang, He, & Wu, 2010). However, the highest added value can be achieved through successful cooperation and congruence of both institutions (Epstein, 2011).

### Strengths and limitations

The sample consists of students from families of higher socio‐economic status in well‐equipped Catholic schools. Although there are findings that deny differences in familial practices based on origin (Moroni, Dumont, & Trautwein, 2016), future studies should include children from less affluent backgrounds, as well as students in schools on lower educational tracks. The relatively high homogeneity of the students in this study, in terms of parents’ educational background and the schools they attend, may have helped to mask the effects of structural conditions, despite the fact that we chose to compare two extreme groups. Therefore, a broader range of structural features could be beneficial in order to confirm or expand the results of the present study.

The survey took place just a few weeks after the students’ transition to secondary school, so their perceptions of intra‐scholastic variables could be distorted. This lack of student experiences in the setting contributed to the decision against the multilevel approach. Nevertheless, it is necessary to mention that the structure of the data is nested, even if the *ICC* is rather low. This aspect should be addressed in future research.

Furthermore, some scholastic and individual influences that may have great impact on scholastic well‐being (e.g., achievement emotions, emotion regulation, and teacher–student interaction; Schlesier, 2020; Schlesier, Roden, & Moschner, 2019; Somerville & Whitebread, 2019) were not included in this study. Likewise, although different types of relationships in class (peer relations and teacher–pupil relations) were not addressed in detail, they were at least measured globally using the pupils’ perceptions of the student‐orientated climate in class (Lenske, 2013). The overall perception of relationships in class may not be wholly trustworthy because students were interrogated just a few weeks after school transition. In order to confirm the results, the model should be further tested with more experienced students in higher grades. A greater sense of belonging and extended social experiences in class should lead to higher effects of this variable on scholastic well‐being.

Finally, the present study tested extreme groups in terms of scholastic well‐being. This design facilitates the aim of discriminant analysis to find variables that differ significantly for groups that are divided in advance of the analyses. Furthermore, it provides a targeted view of perceptions of students with highest and lowest levels of scholastic well‐being, thus enabling the comparison of these two groups in terms of their contexts. The middle third of students – characterized by moderate values of scholastic well‐being – could perceive good conditions in one of the contexts that might mitigate negative effects in the other context. Thus, further research should be conducted to confirm the model based on a sample that is less disjointed.

### Conclusions

In contrast to previous studies (Grigoryeva & Shamionov, 2014; Joronen & Astedt‐Kurki, 2005; Kutsyuruba et al., 2015; Rask, Astedt‐Kurki, Paavilainen, & Laippala, 2003; Suldo & Fefer, 2013), the aim of the current study was to confirm group differences between students who are characterized by an extreme high or low level of scholastic well‐being according to their perceptions of their scholastic and familial environments. As expected, the two groups of students who were preliminarily divided with respect to their level of scholastic well‐being showed significant mean differences in self‐assessed conditions in school and family contexts. Thus, the chosen discriminant variables are suitable for classifying students into two groups and distributing new students into one of the two groups with a high scoring probability.

Since scholastic well‐being is an aspect of increasing interest (Choi, 2018; OECD, 2015, 2018) and of high practical relevance (Borgonovi & Pál, 2016; Morinaj & Hascher, 2019), the present study *offers further possibilities to examine actual states of scholastic well‐being, without the need for additional measures*.

## Conflicts of interest

All authors declare no conflict of interest.

## Author contribution

**Ramona Obermeier:** Conceptualization (equal); Data curation (equal); Formal analysis (equal); Investigation (equal); Methodology (equal); Supervision (equal); Visualization (equal); Writing – original draft (equal); Writing – review & editing (equal). **Juliane Schlesier:** Supervision (equal); Writing – review & editing (equal). **Michaela Gläser‐Zikuda:** Supervision (equal); Writing – review & editing (equal).

## Data Availability

The data that support the findings of this study are available on request from the corresponding author. The data are not publicly available due to privacy or ethical restrictions.

## Footnotes

^1^
International Standard Classification of Education.
